# PET/CT Imaging for Personalized Management of Infectious Diseases

**DOI:** 10.3390/jpm11020133

**Published:** 2021-02-16

**Authors:** Jordy P. Pijl, Thomas C. Kwee, Riemer H. J. A. Slart, Andor W. J. M. Glaudemans

**Affiliations:** 1 Departments of Radiology, Nuclear Medicine and Molecular Imaging, University of Groningen, 9700 RB Groningen, The Netherlands; t.c.kwee@umcg.nl (T.C.K.); r.h.j.a.slart@umcg.nl (R.H.J.A.S.); a.w.j.m.glaudemans@umcg.nl (A.W.J.M.G.); 2 Department of Biomedical Photonic Imaging, Faculty of Science and Technology, University of Twente, 7500 AE Enschede, The Netherlands

**Keywords:** FDG-PET/CT, infection, bloodstream infection, endocarditis, vascular graft infection, spondylodiscitis, cyst infection, white blood cell scintigraphy, total body PET/CT, radiotracers

## Abstract

Positron emission tomography combined with computed tomography (PET/CT) is a nuclear imaging technique which is increasingly being used in infectious diseases. Because infection foci often consume more glucose than surrounding tissue, most infections can be diagnosed with PET/CT using 2-deoxy-2-[18F]fluoro-D-glucose (FDG), an analogue of glucose labeled with Fluorine-18. In this review, we discuss common infectious diseases in which FDG-PET/CT is currently applied including bloodstream infection of unknown origin, infective endocarditis, vascular graft infection, spondylodiscitis, and cyst infections. Next, we highlight the latest developments within the field of PET/CT, including total body PET/CT, use of novel PET radiotracers, and potential future applications of PET/CT that will likely lead to increased capabilities for patient-tailored treatment of infectious diseases.

## 1. Introduction

Infectious diseases are one of the most common reasons for hospital admission worldwide [1]. Commonly diagnosed infections include pneumonia, urinary tract infection, and bloodstream infection [2]. The diagnosis of most infections is often straightforward based on history taking and physical examination, and complemented with laboratory examinations and conventional (chest) radiography when clinically indicated. Microbiologic cultures and antibiotic sensitivity tests can be performed to enable more targeted treatment with narrow-spectrum antibiotic therapy. In some patients with infectious diseases, however, the infection focus may be difficult to detect with conventional diagnostics. This is especially true in patients with implanted foreign materials and electronic devices, such as artificial valves, vascular stents, prosthetic joints, and pacemakers/ICDs [3]. Positron Emission Tomography (PET) is a functional imaging technique that utilizes properties of specific molecules labeled with positron-emitting isotopes to visualize tissues or processes of interest. PET combined with Computed Tomography (PET/CT) allows for the spatial localization of radiotracer accumulation and simultaneous visualization of anatomy and structural abnormalities. PET/CT can be used for a wide variety of diseases, including infections. The most commonly used PET tracer for evaluating infectious diseases is 2-deoxy-2-[18F]fluoro-D-glucose (FDG). FDG-PET/CT can be used to visualize glucose uptake throughout the whole body in a single noninvasive examination. White blood cells and other inflammatory cells that are drawn to infection foci have a high glucose metabolism compared to other cells. Additionally, inflammatory mediators cause a local upregulation of glucose transporters, resulting in an increased cellular FDG uptake. Therefore, infection sites are often readily visible on FDG-PET/CT, even before gross structural changes such as abscess formation have occurred [4].

PET/CT capacity and patient throughput is relatively lower than that of conventional diagnostics such as ultrasonography, CT, and even magnetic resonance imaging (MRI) in most hospitals. Because more time is necessary for patient preparation and scanning, the use of FDG-PET/CT is usually reserved for specific infections in which conventional diagnostics are of limited value for identifying the infection focus [5,6]. Nevertheless, research on the diagnostic role of FDG-PET/CT for various infectious diseases is rapidly expanding leading to more standard application of this technique in a number of infectious diseases [7,8]. In this review, we describe the most common diagnostic applications of FDG-PET/CT in infectious diseases, highlight alternative nuclear imaging techniques used for diagnosing infectious diseases, and discuss the latest developments in the field of PET/CT that will likely contribute to increased capabilities for patient-tailored treatment of infectious diseases.

## 2. Current Common Applications of FDG-PET/CT

### 2.1. Bloodstream Infection of Unknown Origin 

**Figure 1:**
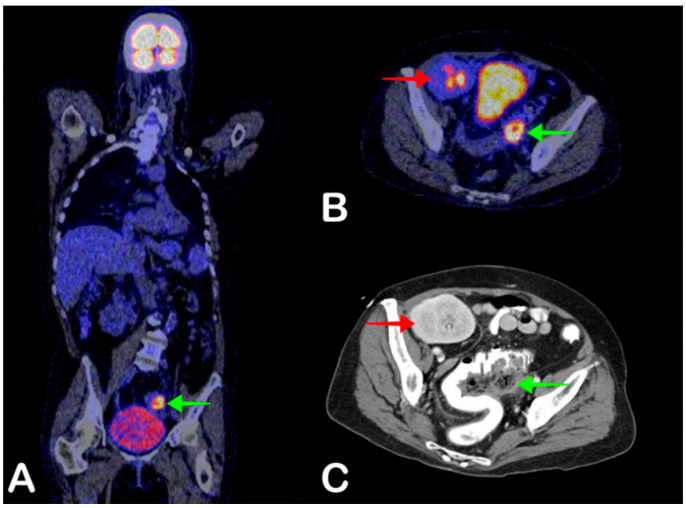
A 54-year-old woman was admitted to the hospital with fever. During hospitalization, she repeatedly had blood cultures positive for *Escherichia coli* and *Klebsiella pneumoniae* without localizing symptoms. Two years earlier, she had received a kidney transplant. Fused coronal2-deoxy-2-[18F]fluoro-D-glucose Positron Emission Tomography combined with Positron Tomography (FDG-PET/CT) showed increased FDG uptake at the left side of the sigmoid colon (**A**, green arrow), also shown on fused axial FDG-PET/CT (**B**, green arrow), indicative of either diverticulitis or abscess formation, as an explanation for the positive blood culture (*Escherichia coli*). Antibiotic treatment was started, but 9 days after PET/CT, the patient developed severe abdominal pain, and abdominal CT was performed. Axial CT also shows the abscess, with adjacent free intraperitoneal air, in keeping with perforated diverticulitis (**C**, green arrow). The kidney transplant in the right iliac fossa is also visible (**B** and **C**, red arrow). A laparotomic sigmoidectomy was performed, after which the patient recovered.

Bloodstream infection, defined as the presence of viable bacteria or fungi in the blood, is a common reason for hospitalization [9] (Figure 1). The most important treatment for bloodstream infection consists of source control and antimicrobial treatment [10]. In patients with an unknown source of infection or with multiple potential infection foci, FDG-PET/CT has been shown to be valuable in diagnosing the infection focus and thereby enabling more targeted treatment [11]. In several large clinical studies, FDG-PET/CT was able to identify the primary infection focus in 56% to 68% of patients with bloodstream infection of unknown origin, and previously unidentified septic emboli in approximately 60% of patients with *Staphylococcus aureus* bacteremia [4,12,13,14,15]. Because timely identification of the infection focus is associated with higher survival rates and is also cost-efficient in, for example, *Staphylococcus aureus* bacteremia, the clinical use of FDG-PET/CT in bloodstream infection of unknown origin is increasing [16,17,18].

In addition to the identification of the primary infection focus, FDG-PET/CT is increasingly performed as a routine examination in patients with a known source of bacteremia that has a high risk of septic dissemination. Risk factors for this include bacteremia with *Staphylococcus aureus*, persisting positive blood cultures despite appropriate antibiotic treatment, the presence of foreign materials such as artificial cardiac valves or vascular stents, electronic devices, or persisting fever [4,14]. Because septic dissemination of a primary infection focus can occur throughout the body and the location of these septic infection foci may not be clinically apparent, FDG-PET/CT is usually the method of choice to examine the whole body for septic infection foci in a single procedure [4]. Additionally, the presence or absence of septic foci also has consequences for the duration of antibiotic treatment. For example, in case of *Staphylococcus aureus* bacteremia, patients are generally treated with antibiotics for two weeks in case of uncomplicated bacteremia. Dissemination of disease is a feature of complicated bacteremia which requires prolonged antibiotic treatment of four to six weeks [19]. Therefore, the results of FDG-PET/CT can be helpful in determining the appropriate duration or adaption of antibiotic treatment.

### 2.2. Fever of Unknown Origin

**Figure 2:**
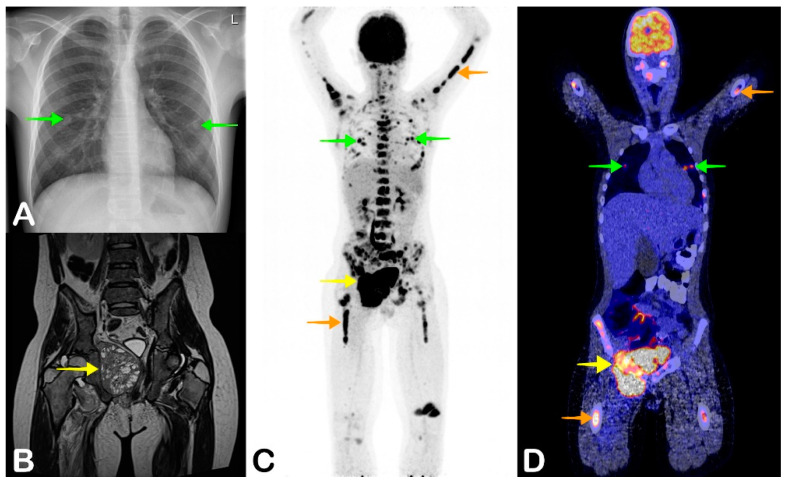
A 13-year-old boy presented to the hospital with a fever of three weeks, weight loss, and general malaise. He also had nonspecific pain in his legs and back and complained of nausea and vomiting. On physical examination, hepatomegaly was noticed. Laboratory testing showed normocytic anemia with a hemoglobin level of 5.2 mmol/L, leukopenia with a leukocyte count of 0.8 × 10^9^/L, and elevated lactate dehydrogenase of 300 U/L. Abdominal ultrasonography confirmed the hepatomegaly, but showed no clear cause of the fever. A chest X-ray showed multiple pulmonary nodules (**A**, green arrows), suggestive of metastases of an unknown primary tumor. Because the patient had multiple nonguiding symptoms, FDG-PET/CT was performed. Coronal maximum intensity projection FDG-PET and fused FDG-PET/CT showed a large FDG-avid mass in the right lesser pelvis (**C** and **D**, yellow arrow), multiple pulmonary metastases (**C** and **D**, green arrows), and focally FDG avid bone marrow (**C** and **D**, orange arrows). Biopsy confirmed the diagnosis of metastasized Ewing sarcoma. MRI was performed before treatment was started, which also showed the primary site of the Ewing sarcoma in the right side of the pelvis (**B**, yellow arrow). After chemotherapy, radiotherapy, and an autologous stem cell transplant, the patient recovered and remained in complete remission for five years of follow-up.

Fever, defined by an elevated body temperature of 38.3 degrees Celsius or higher, is a common symptom that can be caused by a large number of diseases [20] (Figure 2). The causes of fever can usually be categorized into infectious disease, noninfectious inflammatory disease, and malignant disease [21,22]. In some patients, diagnosing the cause of fever can present a diagnostic challenge. Although many different definitions are available, patients are usually considered to have fever of unknown origin when they have persistent fever for two to three weeks with no apparent cause after extensive clinical evaluation [21].

Because elevated glucose uptake occurs in many infectious, inflammatory, and malignant diseases, FDG-PET/CT can be an important aid in diagnosing the cause of the fever. A large number of studies has already been conducted to evaluate the use of FDG-PET/CT in finding the cause of fever. A recent study from 2017 described 15 retrospective studies including a total of 823 patients with fever of unknown origin and one prospective study including 48 patients [22]. In 58% of all patients, the FDG-PET/CT result was considered helpful, which was most often defined by identifying the cause of fever or guiding further therapy [22]. This is in line with a study of our own including 110 children with fever of unknown origin who underwent FDG-PET/CT, in whom the cause of fever was eventually identified in 62% [23]. In 48% of children, this was based on FDG-PET/CT. The most common causes of fever included endocarditis, systemic juvenile idiopathic arthritis, and inflammatory bowel disorder. Diseases such as Kawasaki arteritis, drug-induced fever, familial Mediterranean fever, and urinary tract infection were based on diagnostics other than FDG-PET/CT. Because FDG-PET/CT is able to identify the cause of fever in approximately half of patients with fever of unknown origin, clinicians should consider performing this technique in patients with persistent fever without apparent cause after extensive clinical evaluation. Performing FDG-PET/CT earlier on in patients with persistent fever of unknown origin may also be cost-effective, as it can lead to a faster diagnosis, reduce the number of diagnostic tests, and decrease hospitalization days [24,25].

### 2.3. Infective Endocarditis

**Figure 3:**
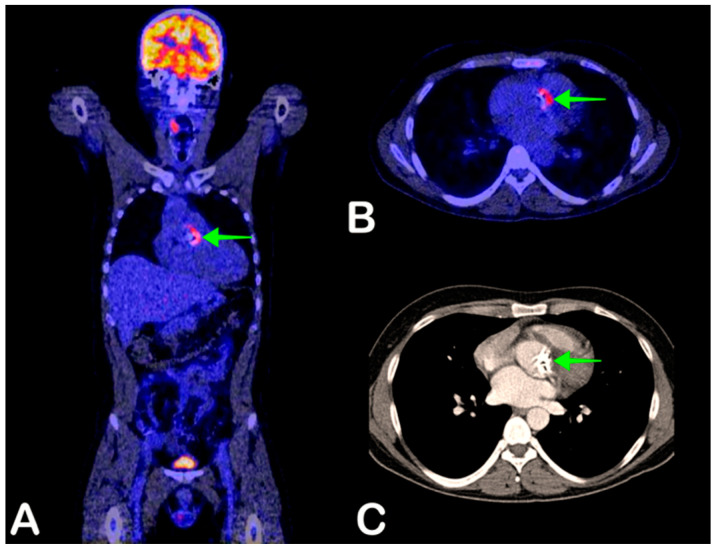
A 39-year-old man presented with general malaise for one week, fever of 41 degrees Celsius, and petechiae. Blood cultures were positive for *Streptococcus pneumoniae*. One year earlier, his native aortic valve was replaced with a mechanical valve. Endocarditis was strongly suspected, but transthoracic and transesophageal ultrasound did not prove valvular vegetations or other signs of infection. Fused coronal and axial FDG-PET/CT showed FDG avidity of the prosthetic aortic valve suggestive of infection (**A** and **B**, green arrow). No other infection foci were found. The aortic valve is also shown on full-dose thoracic CT (**C**, green arrow), without any anatomical signs of infection. The aortic valve was surgically replaced after which the patient recovered.

Infective endocarditis is an endocardial infection with high morbidity and mortality [26] (Figure 3). Although artificial heart valves are an important risk factor for developing infective endocarditis, infective endocarditis also occurs in patients with native valves [27]. Currently, infective endocarditis is mostly diagnosed based on the modified Duke criteria. This diagnostic algorithm consists of major and minor criteria that represent common features of infective endocarditis. The sensitivity of these criteria for diagnosing infective endocarditis is approximately 72% [28]. One of the major criteria is the presence of valvular vegetations on transthoracic or transesophageal ultrasonography. However, the image quality of ultrasonography can be limited for various reasons such as the presence of artificial intracardiac material including artificial heart valves or vascular stents [29]. Because a definitive diagnosis of endocarditis has important treatment consequences such as prolonged intravenous use of antibiotics or even cardiac valve replacement, additional imaging such as FDG-PET/CT or alternatively white blood cell scintigraphy is often performed. Although FDG-PET/CT has a relatively low sensitivity of approximately 14% in patients with native valve endocarditis, it has a high sensitivity of 71–100% in patients with prosthetic valve endocarditis [30,31]. Nevertheless, FDG-PET/CT can still be valuable in native valve endocarditis for the detection of septic infection foci [30]. In current European Society of Cardiology guidelines for the management of infective endocarditis, FDG-PET/CT is only included as a diagnostic technique in patients with suspected, but not definite, prosthetic valve endocarditis [32]. However, a recent study also mentions a role for FDG-PET/CT in patients with definite prosthetic valve endocarditis to detect silent embolism and metastatic infection [33]. In addition, performing FDG-PET/CT seems cost-effective in patients with Gram positive bacteremia and risk factors for septic dissemination, including prosthetic valves. Therefore, a wider implementation of FDG-PET/CT may be recommended in future endocarditis guidelines [18,34].

To increase the sensitivity of FDG-PET/CT for diagnosing endocarditis, adequate patient preparation is very important. Because myocardial tissue is highly glucose metabolic, patients should be kept on a low carbohydrate diet at least 24 h prior to FDG-PET/CT to suppress physiologic FDG uptake of the heart as this masks pathologic FDG uptake of the valves in case of endocarditis [35].

In patients with an infected prosthetic heart valve, choosing between conservative treatment with antibiotics instead of surgical valve replacement can be a difficult decision. In case conservative antibiotic treatment is chosen, follow-up FDG-PET/CT may be performed to assess persisting or resolved FDG avidity of the infected valve, monitor septic complications, and the potential need for surgical replacement.

### 2.4. Vascular Graft Infection

**Figure 4:**
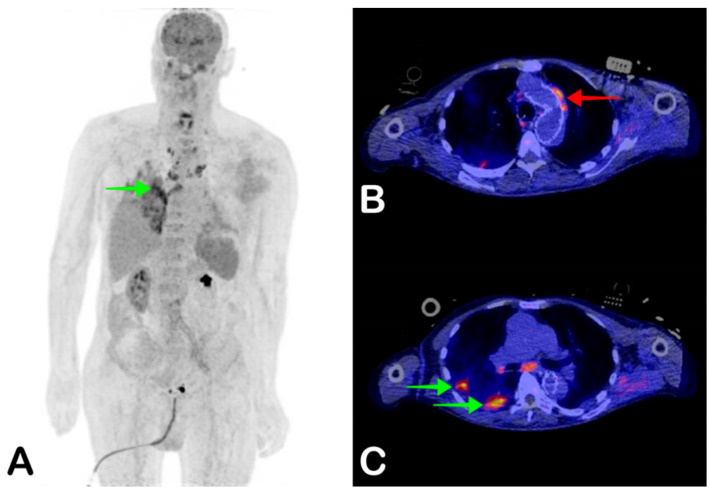
A 72-year-old man with a vascular graft in the aortic arch, abdominal aorta, and left common iliac artery was admitted with a fever of 38.5 degrees Celsius. Blood cultures were positive for *Enterococcus faecalis*. A clinical diagnosis of endocarditis was made based on the modified Duke criteria, although transthoracic and transesophageal ultrasound did not show signs of endocarditis. Coronal maximum-intensity projection FDG-PET showed increased FDG uptake in the right lower pulmonary lobe suggestive of infection (**A**, green arrow), and multiple metabolically active mediastinal and paratracheal lymph nodes. Fused axial FDG-PET/CT showed increased FDG uptake of the aortic arch stent suggestive of infection (**B**, red arrow), as well as FDG avid lesions suggestive of pulmonary infection (**C**, green arrows). The patient died three days after the PET/CT.

Approximately 1% to 5% of patients with a vascular graft develop a vascular graft infection [36] (Figure 4). This can occur shortly after vascular graft implantation (within two months of surgery) which is defined as an early infection, or after more than two months after surgery, which is defined as a late infection [36].

Clinical signs of vascular graft infection include fever, pain located at the vascular graft, erythema around the surgical site, and positive blood cultures. CT angiography may show periprosthetic infiltration or abscess and the formation of gas bubbles in cases of vascular graft infection. To diagnose early vascular graft infection, CT angiography is usually preferred over FDG-PET/CT because postsurgical or foreign body inflammation may also result in elevated FDG uptake around the vascular graft, resulting in false positive results [37]. In late vascular graft infections, however, FDG-PET/CT is the imaging modality of choice due to superior sensitivity (95% for FDG-PET/CT versus 67% for CT angiography), with a specificity of 85% [38,39]. Additionally, the whole body can be assessed for septic infection foci, infected lymph nodes, or another primary focus of infection in case the vascular graft infection is secondary to another infection. Although CT angiography often remains the first step imaging in suspected vascular graft infection, FDG-PET/CT is now included in the standard imaging workflow in suspected vascular graft infection and should especially be considered when CT angiography shows inconclusive results or no signs of infection, while vascular graft infection is still clinically suspected [40].

Diagnosing a vascular graft infection always has major treatment consequences. Because of biofilm formation on the vascular graft, antibiotic therapy will rarely lead to complete eradication of the pathogen. The vascular graft either has to be surgically explanted, or the patient has to be treated with lifelong antibiotics. In patients with another primary focus of infection and only secondary infection of the vascular graft, antibiotic therapy may be sufficient to eliminate the pathogen [40]. Therefore, FDG-PET/CT can present valuable information in patients with suspected vascular graft infection.

### 2.5. Spondylodiscitis

**Figure 5:**
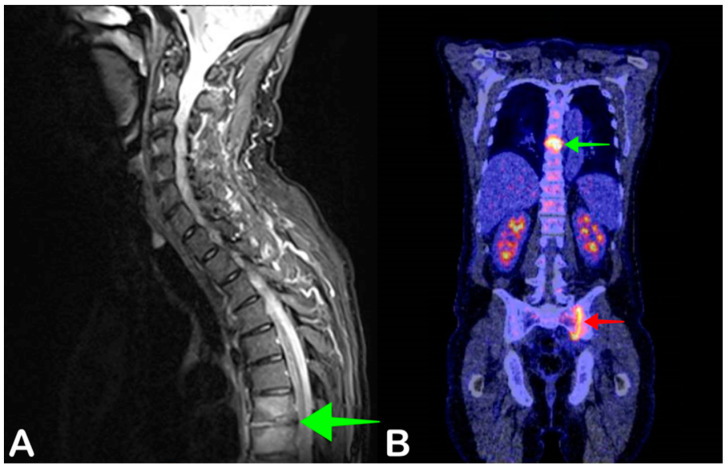
A 63-year-old man presented with fever and back pain. His left upper leg was also painful. Blood cultures were positive for *Staphylococcus aureus.* Sagittal fat-suppressed T2-weighted MRI showed increased signal intensity of thoracic vertebrae seven and eight, and the seventh and eighth intervertebral discs of the thoracic vertebrae–disc complex confirming the spondylodiscitis found on MRI (**A**, green arrow), but also showed increased FDG uptake at the left sacroiliac joint, indicative of sacroiliitis (**B**, red arrow). Antibiotic therapy was continued for six weeks after which the patient recovered.

Spondylodiscitis, including discitis and vertebral osteomyelitis, is a spinal infection that usually presents with back or neck pain, fever, and elevated serum inflammatory markers when the onset is acute (Figure 5). Because of these nonspecific signs, additional imaging is required to diagnose spondylodiscitis. MRI is usually the first modality of choice, with a reported sensitivity of between 67% and 96% for diagnosing spondylodiscitis [41]. The sensitivity of MRI largely depends on the stage of disease, as MRI has a lower sensitivity in patients with an early infection. Early diagnosis and adequate treatment are very important for treatment outcome, as a delayed diagnosis may lead to complications such as persisting back pain or even paraplegia [42].

Although MRI is often the modality of choice in patients with suspected spondylodiscitis, it can present important limitations in patients with foreign spinal materials (after spinal osteosynthesis, for example) causing scatter artefacts on MRI, which significantly decreases image readability. Some patients may also not be eligible for MRI due to non-MRI compatible implants or claustrophobia. In these patients, FDG-PET/CT is often used to examine patients with suspected spondylodiscitis. As FDG-PET/CT detects elevated glucose uptake associated with infection and does not rely on anatomical changes, it may also show higher sensitivity in early spondylodiscitis compared to MRI [43].

The reported sensitivity of FDG-PET/CT for diagnosing spondylodiscitis is 96%, with a specificity of 95% [41]. Because treatment for spondylodiscitis includes antibiotic treatment for at least six weeks, a definite diagnosis is very important to ensure an adequate duration of treatment, as antibiotics may be prescribed for a shorter duration and a lower dose when no infection focus is found on MRI or other diagnostics [42]. Follow-up FDG-PET/CT after a diagnosis of spondylodiscitis may also be performed to evaluate response to antibiotic treatment.

### 2.6. Cyst Infection

**Figure 6:**
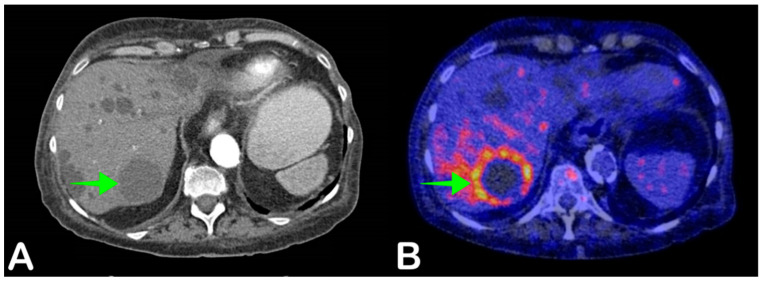
A 68-year-old woman presented with general malaise and night sweats for two weeks. She did not have a fever. She had received a kidney transplant 20 years earlier due to kidney failure caused by autosomal dominant polycystic kidney disease (ADPKD). Blood cultures were positive for *Escherichia coli*. Abdominal CT showed multiple liver cysts consistent with ADPKD, but no obvious signs of infection (**A**, green arrow). Fused axial FDG-PET/CT demonstrated increased FDG uptake in a large cyst in liver segment VII, suggestive of infection (**B**, green arrow). Antibiotic therapy was started with ciprofloxacin, after which the patient recovered.

In patients with multiple abdominal cysts, including patients with autosomal dominant polycystic kidney disease (ADPKD) and polycystic liver disease (PLD), cyst infection can present a diagnostic challenge (Figure 6). Patients usually present with nonspecific signs such as abdominal pain and fever [44]. Conventional diagnostics such as ultrasonography or CT often show no signs of infection or nonspecific signs, such as cystic wall thickening [45]. The gold diagnostic standard is cyst puncture to obtain fluid that can be microbiologically cultured. However, this poses a risk of complications such as contamination of adjacent cysts or bleeding [46]. Therefore, percutaneous puncture is usually only performed in case of frank abscess formation, antibiotic treatment failure, or large infected cysts [47].

Achieving a definitive diagnosis is important for treatment, as cyst infections usually require specific antibiotic treatment with lipophilic antibiotics that can penetrate cyst walls, such as fluoroquinolones. Empirical antibiotic treatment is usually not suitable for treating cyst infections [44]. FDG-PET/CT has been shown to be valuable in diagnosing cyst infection, especially in patients with multiple cysts, with a reported sensitivity of 77–100% and specificity of 75–100% in patients with ADPKD [48,49,50,51]. When cyst infection is suspected, performing FDG-PET/CT early in the diagnostic workup can lead to faster diagnosis and subsequent faster initiation of adequate antibiotic treatment.

## 3. Limitations of FDG-PET/CT

Because most cells metabolize glucose for ATP synthesis, physiologic FDG uptake occurs throughout the body. Therefore, FDG uptake is not specific for infection. Besides infection, FDG uptake can be locally elevated for other reasons as well [8]. For example, tumors often show increased FDG uptake, inflammatory diseases such as vasculitis or rheumatoid arthritis can cause increased FDG uptake, and postsurgical inflammation can also present challenges in diagnosing infection in patients who recently underwent a surgical procedure [52]. Additionally, foreign materials such as prosthetic joints, prosthetic heart valves, or vascular grafts often cause mild sterile inflammation and subsequent FDG uptake, affecting FDG-PET/CT’s sensitivity and specificity for diagnosing infection [53]. Furthermore, not all infections can be diagnosed with FDG-PET/CT. For example, urinary tract infections may be difficult to diagnose due to renal FDG excretion.

Although conventional imaging such as CT or ultrasonography can often readily be performed, FDG-PET/CT requires adequate patient preparation. For example, patients have to fast for at least 6 h before FDG-PET/CT is performed, and refrain from carbohydrate-rich foods and (intravenous) fluids for at least 24 h when the possibility of endocarditis needs to be evaluated [7]. This can dissuade physicians from performing FDG-PET/CT in acutely ill patients, especially when FDG-PET/CT is only performed during office hours. Hospital capacity for FDG-PET/CT is usually also much lower than for stand-alone CT.

Antibiotic treatment may also negatively affect FDG-PET/CT’s ability to diagnose infection. In a study of our own, longer duration of antibiotic treatment was associated with a lower chance of detecting an infection focus on FDG-PET/CT in patients with bloodstream infection [15]. However, another study from 2017 found no significant effect between duration of antibiotic treatment and diagnosing infection on FDG-PET/CT [54]. It could be hypothesized that longer antibiotic treatment may clear the infection before FDG-PET/CT is performed, but future research is needed to confirm this. Nevertheless, FDG-PET/CT should not be delayed in patients with bloodstream infection of unknown origin, especially not when there are risk factors for septic dissemination [4].

## 4. White Blood Cell Cintigraphy

In some patients, radiolabeled white blood cells may be used to overcome these limitations, as the accumulation of leukocytes is generally more specific for infection than increased glucose uptake. White blood cell (WBC) scintigraphy is performed on a gamma camera. A common indication for WBC scintigraphy is suspected prosthetic joint infection. However, this is also dependent on the location of the infection. For example, in patients with a prosthetic hip infection, elevated FDG uptake due to sterile inflammation is expected around the head and neck of the prosthesis, but not at other sites [55]. Extensive, heterogeneous FDG uptake along the bone prosthesis interface, especially in the middle portion of the shaft, is indicative of periprosthetic infection. White blood cell scintigraphy is sometimes also performed in patients with suspected endocarditis or vascular graft infection, especially when results from FDG-PET/CT are inconclusive but clinical suspicion of infection remains.

Several tracers can be used to radiolabel leukocytes. One of the most commonly used tracers is Technetium 99 m. Because this radioisotope does not produce positrons but gamma radiation photons, Single-Photon Emission Computed Tomography (SPECT) is used for this procedure instead of PET. PET-tracers can also be used to label leukocytes. Because labelled leukocytes require 20–24 h to be recruited to infection sites, FDG is not suitable for radiolabeling glucose due to its short half-life [56].

WBC scintigraphy also presents important limitations compared to FDG-PET/CT. The procedure is much more complex than regular FDG-PET/CT, the resolution of SPECT is inferior to the resolution of PET, and WBC scintigraphy also presents logistical challenges. First, blood has to be drawn from patients to obtain autologous leukocytes. Then, the harvested leukocytes have to be radiolabeled and reinjected into the patient, which also poses a risk of blood mix-up with other patients. Usually, two scans at two different time intervals are necessary so they can be compared to be able to accurately diagnose infection. The whole procedure usually takes two days to complete. These factors often render white blood cell scintigraphy less favorable than FDG-PET/CT.

## 5. Future Applications of PET/CT

### 5.1. Total Body PET/CT

One of the most promising current advances in PET/CT technology is the development of total body PET/CT [57]. Current PET/CT systems mostly operate with a 20 cm wide detector ring and thus a maximum of 20 cm field of view per bed position. To image the whole body, the table is shifted through the detector ring while the patient has to lie still during the entire procedure. For PET imaging of the whole body, a short field of view presents important limitations. When positron annihilation occurs, two anticollinear high energy photons are ejected in a random direction. As FDG is distributed through the whole body, only 3–5% of the high energy photons emitted hit the detector ring and are recorded, and the photons emitted outside the 20 cm field of view are not recorded. This means that less than 1% of the positron annihilation events that occur in the patient during PET scanning are recorded on current PET/CT systems [58].

The new total body PET/CT systems try to overcome this limitation by significantly extending the field of view. Total body PET/CT systems have an extended field of view of up to 200 cm instead of 20 cm. During scanning, the patient will be placed in this 200 cm tube covered by PET detectors (or slightly shorter tube length, depending on the model), which will significantly decrease photon scattering outside the field of view. For a 200 cm total body PET/CT system, this could theoretically increase sensitivity by a factor of 40 compared to whole-body scans on current PET/CT systems with similar scanning time and FDG dosage. Alternatively, it could also decrease scanning time by a factor of 40 to 15–30 s with similar sensitivity and FDG dosage, or decrease FDG dosage by a factor of 40 to 0.2 mSv (equivalent to 2 chest X-rays) while maintaining similar sensitivity and scanning time [58,59]. The ideal settings and properties of these total body PET/CT systems will be depending on the type of examination and clinical experience, but it will likely revolutionize the possibilities of PET/CT. For infectious disease, it would likely be possible to detect much smaller metastatic infection foci, increase the possibilities of follow-up PET/CT imaging to monitor treatment response, increase the number of infection-specific radiotracers that can be effectively used in PET imaging, and enable real time imaging of various organ–organ axes. Severely ill patients can be scanned very rapidly, and this will benefit intensive care patients.

### 5.2. Infection-Specific Radiotracers

FDG is the most widely used radiotracer for diagnosing infectious disease with PET/CT. As already mentioned, elevated FDG uptake due to infection cannot always be distinguished from elevated FDG uptake due to sterile inflammation or due to postsurgical inflammation. Therefore, many studies have been performed and are still ongoing to develop more infection-specific radiotracers [60,61,62]. These include radiolabeled bacteria-specific monoclonal antibodies such as antibodies against *Staphylococcus aureus* surface molecule lipoteichoic acid labeled with Zirkonium-89 [63], radiolabeled antibiotics such as ciprofloxacin or fluoropropyl-trimethoprim labeled with Fluorine-18 [64,65] radiolabeled antimicrobial peptides such as ubiquicidin labeled with Gallium-68 [66], radiolabeled molecules involved in bacteria-specific synthesis pathways such as p-aminobenzoic acid labeled with Fluorine-18 [67], and fluorodeoxysorbitol labeled with Fluorine-18 which specifically targets Enterobacteriaceae [68] (Table 1). Most of these novel tracers have only been used in vitro, in animal models, or in small human-based research settings. Nevertheless, the clinical application of bacteria or infection-specific radiotracers could present important benefits compared to current diagnostic possibilities. In patients with nonspecific signs of prosthetic joint infection, for example, being able to distinguish aseptic loosening from infection could prevent invasive procedures such as repeated biopsies (which also pose a risk of infecting the prosthesis) or unnecessary surgical replacement of the prosthesis. In cases where biopsy may pose significant risks due to the anatomic location of the lesion of interest, bacteria-specific tracers may be used to identify the specific bacterial strain and subsequently enable more specific antimicrobial treatment.

The half-life of the isotope used in radiotracers has various implications. For medical practices, isotopes with a short half-life, such as FDG with a half-life of 110 min, are often preferred due to lower radiation exposure [70]. In most examinations such as FDG-PET/CT, FDG quickly reaches the target cells and PET/CT is often performed one hour after FDG administration. Some radiotracers, however, require more time to reach their target cells. In case of larger molecules such as radiolabeled antibodies, it takes several days for the antibodies to reach their target cells [74]. This requires the use of isotopes with a longer half-life, but is also associated with higher radiation exposure. However, total body PET/CT could significantly decrease this radiation exposure.

Limitations of bacteria-specific radiotracers include the fact that the number of bacteria present in a low-grade infection may be too low for current PET/CT systems to detect and visualize. However, an increased sensitivity of PET/CT with novel PET/CT systems such as total body PET/CT may enable more widespread bacteria-specific PET imaging. Of interest is the fact that alternative techniques to specifically diagnose these infections are also being explored, such as in vivo imaging using fluorescently labeled vancomycin [75].

### 5.3. Personalized Duration of Antibiotic Treatment

Most patients with infectious diseases are treated with antibiotics or antifungals for a standardized time duration, depending on the type and extent of infection, microorganism species, and clinical response to treatment in terms of symptoms and infection parameters such as C-reactive protein and leukocyte count. In uncomplicated infections such as urinary tract infections or mild pneumonia that only require a short duration of antimicrobial treatment without important side effects, a standardized duration of antibiotic treatment can be accepted even though some of those patients may not need to be treated for the standardized duration of time.

Patients with more complicated and difficult to treat infections, such as endocarditis, vascular graft infection, or hernia mesh infection may need to receive antimicrobial treatment for an extended period ranging from multiple weeks to lifelong suppression therapy [76]. These standardized treatment durations are often based on large cohorts of patients with limited attention to patient-specific characteristics. Physicians may be hesitant to deviate from these standardized long-term treatment plans. Not all patients need such prolonged antimicrobial treatment, but guidance is needed [77]. In these patients, it would be very interesting to follow up infection activity either by using PET/CT with FDG or with more infection-specific novel radiotracers. Future research could be aimed at the relation between infectious disease activity on PET/CT and clinical response over the course of treatment, as this may also allow more patient-tailored treatment. Quantitative FDG-PET/CT follow up is already performed in oncologic diseases such as lymphoma and melanoma to monitor treatment response, but may also be beneficial in patients with chronic infection and long-term antimicrobial treatment [78]. Additionally, (follow up) PET/CT may also aid physicians in deciding to switch from intravenous to oral antibiotic treatment, or from more expensive antifungals such as anidulafungin to the cheaper but maybe less effective fluconazole [79].

It is difficult to predict the cost effectiveness of applying such a technique. PET/CT may be a relatively expensive imaging technique, but prolonged hospitalization for intravenous administration of antibiotics or the placement of peripherally inserted central catheters for intravenous antibiotic treatment at home may be more expensive. Additionally, the prolonged use of antibiotics also increases the risk of severe side effects such as *Clostridium difficile* superinfection, bone marrow suppression, or toxic levels of aminoglycosides leading to deafness or kidney failure, which are all associated with significant morbidity costs and reduced quality of life [77].

## 6. Conclusions

PET/CT is a valuable imaging technique that is increasingly being used in infectious diseases. Although it can accurately diagnose a number of infectious diseases, its main benefits are derived from the ability to examine the whole body for an infection focus or septic infection foci in a single procedure, the ability to diagnose early infections before significant anatomical changes have occurred, and the ability to diagnose artificial material infections such as vascular graft infection, orthopedic prostheses, electronic implantable devices, or prosthetic valve endocarditis. Technological developments in PET/CT and tracers will likely contribute to more widespread use of PET/CT in diagnosing and monitoring infectious disease, allowing more patient-tailored treatment of infectious disease in terms of treatment duration and deciding between conservative antibiotic treatment or invasive surgical procedures.

## Figures and Tables

**Table 1:** Various examples of bacterial (novel)tracers for PET/CT.

Tracer	Isotope (Half-Life)	Ligand	Target/Substrate	Comment
89Zr-SAC55	Zirkonium-89 (78.4 h) [69]	Monoclonal antibody	*Staphylococcus aureus* surface molecule lipoteichoic acid	Promising results from animal studies, currently no human studies available [63]
18F-ciprofloxacin	Fluorine-18 (110 min) [70]	Ciprofloxacin	Bacterial DNA gyrase or topoisomerase IV	In-human studies did not indicate bacteria-specific binding [64]
18F-fluoropropyl-trimethoprim	Fluorine-18 (110 min) [70]	Trimethoprim	Bacterial dihydrofolate reductase	No in-human studies available [65]
68Ga-NOTA-UBI	Gallium-68 (68 min) [71]	Chelated ubiquicidin	Bacterial cytoplasmic membrane	Promising results in animal models, but very limited in-human experience [66]
2-18F-F-p-Aminobenzoic Acid	Fluorine-18 (110 min) [70]	*p*-aminobenzoic acid	Dihydropteroate synthase	Promising results from animal studies, also targets different types of bacteria [72]
2-18F-fluorodeoxysorbitol	Fluorine-18 (110 min) [70]	Sorbitol	Sorbitol-6-phosphate dehydrogenase	Promising results from animal studies, limited in-human experience. Sorbitol is not metabolized by Gram positive bacteria [73].

## Data Availability

No new data were created or analyzed in this study. Data sharing is not applicable to this article.

## References

[1] Rudd K.E., Johnson S.C., Agesa K.M., Shackelford K.A., Tsoi D., Kievlan D.R., Colombara D.V., Ikuta K.S., Kissoon N., Finfer S. (2020). Global, regional, and national sepsis incidence and mortality, 1990–2017: Analysis for the Global Burden of Disease Study. Lancet.

[2] Bradley S.F. (2007). Infections. Pract. Geriatr..

[3] Vanepps J.S., Younger J.G. (2016). Implantable Device-Related Infection. Shock.

[4] Vos F.J., Bleeker-Rovers C.P., Sturm P.D., Krabbe P.F.M., Van Dijk A.P.J., Cuijpers M.L.H., Adang E.M.M., Wanten G.J.A., Kullberg B.-J., Oyen W.J.G. (2010). 18F-FDG PET/CT for Detection of Metastatic Infection in Gram-Positive Bacteremia. J. Nucl. Med..

[5] Vaidyanathan S., Patel C., Scarsbrook A., Chowdhury F. (2015). FDG PET/CT in infection and inflammation—Current and emerging clinical applications. Clin. Radiol..

[6] Fuchs S., Grössmann N., Ferch M., Busse R., Wild C. (2019). Evidence-based indications for the planning of PET or PET/CT capacities are needed. Clin. Transl. Imaging.

[7] Jamar F., Buscombe J., Chiti A., Christian P.E., Delbeke D., Donohoe K.J., Israel O., Martin-Comin J., Signore A. (2013). EANM/SNMMI Guideline for 18F-FDG Use in Inflammation and Infection. J. Nucl. Med..

[8] Kung B.T., Seraj S.M., Zadeh M.Z., Rojulpote C., Kothekar E., Ayubcha C., Ng K.S., Ng K.K., Au-Yong T.K., Werner T.J. (2019). An update on the role of 18F-FDG-PET/CT in major infectious and inflammatory diseases. Am. J. Nucl. Med. Mol. Imaging.

[9] Goto M., Al-Hasan M.N. (2013). Overall burden of bloodstream infection and nosocomial bloodstream infection in North America and Europe. Clin. Microbiol. Infect..

[10] Lagunes L., Encina B., Ramirez-Estrada S. (2016). Current understanding in source control management in septic shock patients: A review. Ann. Transl. Med..

[11] Treglia G. (2019). Diagnostic Performance of 18F-FDG PET/CT in Infectious and Inflammatory Diseases according to Published Meta-Analyses. Contrast Media Mol. Imaging.

[12] Tseng J.-R., Chen K.-Y., Lee M.-H., Huang C.-T., Wen Y.-H., Yen T.-C. (2013). Potential Usefulness of FDG PET/CT in Patients with Sepsis of Unknown Origin. PLoS ONE.

[13] Brøndserud M.B., Pedersen C., Rosenvinge F.S., Høilund-Carlsen P.F., Hess S. (2019). Clinical value of FDG-PET/CT in bacteremia of unknown origin with catalase-negative gram-positive cocci or Staphylococcus aureus. Eur. J. Nucl. Med. Mol. Imaging.

[14] Berrevoets M.A., Kouijzer I.J., Aarntzen E.H., Janssen M.J., Wertheim H.F., Kullberg B.-J., Oyen W.J.G., Bleeker-Rovers C.P., De Geus-Oei L.-F., Oever J.T. (2017). 18 F-FDG PET/CT Optimizes Treatment in Staphylococcus Aureus Bacteremia and Is Associated with Reduced Mortality. J. Nucl. Med..

[15] Pijl J., Glaudemans A., Slart R., Yakar D., Wouthuyzen-Bakker M., Kwee T. (2018). FDG-PET/CT for Detecting an Infection Focus in Patients with a Bloodstream Infection: Factors Affecting Diagnostic Yield. SSRN Electron. J..

[16] Tabah A., Koulenti D., Laupland K.B., Misset B., Valles J., De Carvalho F.B., Paiva J.A., Çakar N., Ma X., Eggimann P. (2012). Characteristics and determinants of outcome of hospital-acquired bloodstream infections in intensive care units: The EUROBACT International Cohort Study. Intensiv. Care Med..

[17] Hess S., Hansson S.H., Pedersen K.T., Basu S., Høilund-Carlsen P.F. (2014). FDG-PET/CT in Infectious and Inflammatory Diseases. PET Clin..

[18] Vos F.J., Bleeker-Rovers C.P., Kullberg B.J., Adang E.M., Oyen W.J.G. (2011). Cost-Effectiveness of Routine 18F-FDG PET/CT in High-Risk Patients with Gram-Positive Bacteremia. J. Nucl. Med..

[19] Holland T.L., Arnold C.J., Fowler V.G. (2014). Clinical Management of Staphylococcus aureus Bacteremia. JAMA.

[20] Petersdorf R.G., Beeson P.B. (1961). Fever of unexplained origin: Report on 100 cases. Medicine.

[21] Meller J., Sahlmann C.-O., Scheel A.K. (2007). 18F-FDG PET and PET/CT in fever of unknown origin. J. Nucl. Med..

[22] Kouijzer I.J., Mulders-Manders C.M., Bleeker-Rovers C.P., Oyen W.J. (2018). Fever of Unknown Origin: The Value of FDG-PET/CT. Semin. Nucl. Med..

[23] Pijl J.P., Kwee T.C., Legger G., Peters H.J., Armbrust W., Schölvinck E., Glaudemans A.W. (2020). Role of FDG-PET/CT in children with fever of unknown origin. Eur. J. Nucl. Med. Mol. Imaging.

[24] Nakayo E.B., Vicente A.G., Castrejón Á.M.S., Narváez J.M., Rubio M.T., García V.P., García J.C. (2012). Analysis of cost-effectiveness in the diagnosis of fever of unknown origin and the role of 18F-FDG PET–CT: A proposal of diagnostic algorithm. Rev. Esp. Med. Nucl. Imagen Mol. Engl. Ed..

[25] Balink H., Tan S.S., Veeger N.J.G.M., Holleman F., Van Eck-Smit B.L.F., Bennink R.J., Verberne H.J. (2015). 18F-FDG PET/CT in inflammation of unknown origin: A cost-effectiveness pilot-study. Eur. J. Nucl. Med. Mol. Imaging.

[26] McDonald J.R. (2009). Acute Infective Endocarditis. Infect. Dis. Clin. N. Am..

[27] Rajani R., Klein J.L. (2020). Infective endocarditis: A contemporary update. Clin. Med..

[28] Shrestha N., Shakya S., Hussain S., Pettersson G., Griffin B., Gordon S. (2017). Sensitivity and Specificity of Duke Criteria for Diagnosis of Definite Infective Endocarditis: A Cohort Study. Open Forum Infect. Dis..

[29] Evangelista A. (2004). Echocardiography in infective endocarditis. Hearth.

[30] Chen W., Dilsizian V. (2018). FDG PET/CT for the diagnosis and management of infective endocarditis: Expert consensus vs evidence-based practice. J. Nucl. Cardiol..

[31] Gomes A., Glaudemans A.W.J.M., Touw D.J., Van Melle J.P., Willems T.P., Maass A.H., Natour E., Prakken N.H.J., Borra R.J.H., Van Geel P.P. (2017). Diagnostic value of imaging in infective endocarditis: A systematic review. Lancet Infect. Dis..

[32] Habib G., Lancellotti P., Antunes M.J., Bongiorni M.G., Casalta J.-P., Del Zotti F., Dulgheru R., El Khoury G., Erba P.A., Iung B. (2016). 2015 ESC Guidelines for the management of infective endocarditis. The Task Force for the Management of Infective Endocarditis of the European Society of Cardiology (ESC). Endorsed by: European Association for Cardio-Thoracic Surgery (EACTS), the European Association of Nuclear Medicine (EANM). G. Ital. Cardiol. Rome.

[33] Erba P.A., Pizzi M.N., Roque A., Salaun E., Lancellotti P., Tornos P., Habib G. (2019). Multimodality Imaging in Infective Endocarditis. Circulation.

[34] Bleeker-Rovers C.P., Vos F.J., Wanten G.J.A., Van Der Meer J.W.M., Corstens F.H.M., Kullberg B.-J., Oyen W.J.G. (2005). 18F-FDG PET in detecting metastatic infectious disease. J. Nucl. Med..

[35] Slart R.H.J.A., Glaudemans A.W.J.M., Gheysens O., Lubberink M., Kero T., Dweck M.R., Habib G., Gaemperli O., Saraste A., Gimelli A. (2020). Procedural recommendations of cardiac PET/CT imaging: Standardization in inflammatory-, infective-, infiltrative-, and innervation (4Is)-related cardiovascular diseases: A joint collaboration of the EACVI and the EANM. Eur. J. Nucl. Med. Mol. Imaging.

[36] Wilson W.R., Bower T.C., Creager M.A., Amin-Hanjani S., O’Gara P.T., Lockhart P.B., Darouiche R.O., Ramlawi B., Derdeyn C.P., Bolger A.F. (2016). Vascular Graft Infections, Mycotic Aneurysms, and Endovascular Infections: A Scientific Statement from the American Heart Association. Circulation.

[37] Keidar Z., Nitecki S. (2013). FDG-PET in Prosthetic Graft Infections. Semin. Nucl. Med..

[38] Keidar Z., Engel A., Hoffman A., Israel O., Nitecki S. (2007). Prosthetic Vascular Graft Infection: The Role of 18F-FDG PET/CT. J. Nucl. Med..

[39] Saleem B.R., Pol R.A., Slart R.H.J.A., Reijnen M.M.P.J., Zeebregts C.J. (2014). 18F-Fluorodeoxyglucose Positron Emission Tomography/CT Scanning in Diagnosing Vascular Prosthetic Graft Infection. BioMed Res. Int..

[40] Chakfé N., Diener H., Lejay A., Assadian O., Berard X., Caillon J., Fourneau I., Glaudemans A.W., Koncar I., Lindholt J. (2020). Editor’s Choice—European Society for Vascular Surgery (ESVS) 2020 Clinical Practice Guidelines on the Management of Vascular Graft and Endograft Infections. Eur. J. Vasc. Endovasc. Surg..

[41] Smids C., Kouijzer I.J.E., Vos F.J., Sprong T., Hosman A.J.F., De Rooy J.W.J., Aarntzen E.H.J.G., De Geus-Oei L.-F., Oyen W.J.G., Bleeker-Rovers C.P. (2017). A comparison of the diagnostic value of MRI and 18F-FDG-PET/CT in suspected spondylodiscitis. Infection.

[42] Gouliouris T., Aliyu S.H., Brown N.M. (2010). Spondylodiscitis: Update on diagnosis and management. J. Antimicrob. Chemother..

[43] Altini C., Lavelli V., Niccoli-Asabella A., Sardaro A., Branca A., Santo G., Ferrari C., Rubini G. (2020). Comparison of the Diagnostic Value of MRI and Whole Body 18F-FDG PET/CT in Diagnosis of Spondylodiscitis. J. Clin. Med..

[44] Torres V.E., Harris P.C., Pirson Y. (2007). Autosomal dominant polycystic kidney disease. Lancet.

[45] Oh J., Shin C.-I., Kim S.Y. (2018). Infected cyst in patients with autosomal dominant polycystic kidney disease: Analysis of computed tomographic and ultrasonographic imaging features. PLoS ONE.

[46] Lantinga M.A., Drenth J.P., Gevers T.J.G. (2014). Diagnostic criteria in renal and hepatic cyst infection. Nephrol. Dial. Transpl..

[47] Lantinga M.A., Casteleijn N.F., Geudens A., De Sévaux R.G., Van Assen S., Leliveld A.M., Gansevoort R.T., Drenth J.P. (2016). Management of renal cyst infection in patients with autosomal dominant polycystic kidney disease: A systematic review. Nephrol. Dial. Transpl..

[48] Sallée M., Rafat C., Zahar J.-R., Paulmier B., Grünfeld J.-P., Knebelmann B., Fakhouri F. (2009). Cyst Infections in Patients with Autosomal Dominant Polycystic Kidney Disease. Clin. J. Am. Soc. Nephrol..

[49] Bobot M., Ghez C., Gondouin B., Sallée M., Fournier P., Burtey S., Legris T., Dussol B., Berland Y., Souteyrand P. (2016). Diagnostic performance of [18F] fluorodeoxyglucose positron emission tomography–computed tomography in cyst infection in patients with autosomal dominant polycystic kidney disease. Clin. Microbiol. Infect..

[50] Jouret F., Lhommel R., Beguin C., Devuyst O., Pirson Y., Hassoun Z., Kanaan N. (2011). Positron-Emission Computed Tomography in Cyst Infection Diagnosis in Patients with Autosomal Dominant Polycystic Kidney Disease. Clin. J. Am. Soc. Nephrol..

[51] Pijl J.P., Glaudemans A.W., Slart R.H., Kwee T.C. (2018). 18F-FDG PET/CT in Autosomal Dominant Polycystic Kidney Disease Patients with Suspected Cyst Infection. J. Nucl. Med..

[52] Garg G., Benchekroun M.T., Abraham T. (2017). FDG-PET/CT in the Postoperative Period: Utility, Expected Findings, Complications, and Pitfalls. Semin. Nucl. Med..

[53] Gelderman S.J., Jutte P.C., Boellaard R., Ploegmakers J.J.W., García D.V., Kampinga G.A., Glaudemans A.W.J.M., Wouthuyzen-Bakker M. (2018). 18F-FDG-PET uptake in non-infected total hip prostheses. Acta Orthop..

[54] Kagna O., Kurash M., Ghanem-Zoubi N., Keidar Z., Israel O. (2017). Does Antibiotic Treatment Affect the Diagnostic Accuracy of18F-FDG PET/CT Studies in Patients with Suspected Infectious Processes?. J. Nucl. Med..

[55] Basu S., Kwee T.C., Saboury B., Garino J.P., Nelson C.L., Zhuang H., Parsons M., Chen W., Kumar R., Salavati A. (2014). FDG PET for Diagnosing Infection in Hip and Knee Prostheses. Clin. Nucl. Med..

[56] Lauri C., Glaudemans A.W.J.M., Campagna G., Keidar Z., Muchnik Kurash M., Georga S., Arsos G., Noriega-Álvarez E., Argento G., Kwee T.C. (2020). Comparison of White Blood Cell Scintigraphy, FDG PET/CT and MRI in Suspected Diabetic Foot Infection: Results of a Large Retrospective Multicenter Study. J. Clin. Med..

[57] Vandenberghe S., Moskal P., Karp J.S. (2020). State of the art in total body PET. EJNMMI Phys..

[58] Cherry S.R., Jones T., Karp J.S., Qi J., Moses W.W., Badawi R.D. (2018). Total-Body PET: Maximizing Sensitivity to Create New Opportunities for Clinical Research and Patient Care. J. Nucl. Med..

[59] Diederich S., Lenzen H. (2000). Radiation exposure associated with imaging of the chest: Comparison of different radiographic and computed tomography techniques. Cancer.

[60] Auletta S., Varani M., Horvat R., Galli F., Signore A., Hess S. (2019). PET Radiopharmaceuticals for Specific Bacteria Imaging: A Systematic Review. J. Clin. Med..

[61] Welling M.M., Hensbergen A.W., Bunschoten A., Velders A.H., Roestenberg M., Van Leeuwen F.W.B. (2019). An update on radiotracer development for molecular imaging of bacterial infections. Clin. Transl. Imaging.

[62] Gordon O., Ruiz-Bedoya C.A., Ordonez A.A., Tucker E.W., Jain S.K. (2019). Molecular Imaging: A Novel Tool to Visualize Pathogenesis of Infections In Situ. mBio.

[63] Pickett J.E., Thompson J.M., Sadowska A., Tkaczyk C., Sellman B.R., Minola A., Corti D., Lanzavecchia A., Miller L.S., Thorek D.L. (2018). Molecularly specific detection of bacterial lipoteichoic acid for diagnosis of prosthetic joint infection of the bone. Bone Res..

[64] Langer O., Brunner M., Zeitlinger M., Ziegler S., Dobrozemsky G., Lackner E., Joukhadar C., Mitterhauser M., Wadsak W., Minar E. (2004). In vitro and in vivo evaluation of [18F]ciprofloxacin for the imaging of bacterial infections with PET. Eur. J. Nucl. Med. Mol. Imaging.

[65] Sellmyer M.A., Lee I., Hou C., Weng C.-C., Li S., Lieberman B.P., Zeng C., Mankoff D.A., Mach R.H. (2017). Bacterial Infection Imaging with [18F]Fluoropropyl-Trimethoprim. Proc. Natl. Acad. Sci. USA.

[66] Ebenhan T., Sathekge M.M., Lengana T., Koole M., Gheysens O., Govender T., Zeevaart J.R., Lenagana T. (2017). 68Ga-NOTA-Functionalized Ubiquicidin: Cytotoxicity, Biodistribution, Radiation Dosimetry, and First-in-Human PET/CT Imaging of Infections. J. Nucl. Med..

[67] Zhang Z., Ordonez A.A., Wang H., Li Y., Gogarty K.R., Weinstein E.A., Daryaee F., Merino J., Yoon G.E., Kalinda A.S. (2018). Positron Emission Tomography Imaging with 2-[18F]F-p-Aminobenzoic Acid Detects Staphylococcus Aureus Infections and Monitors Drug Response. ACS Infect. Dis..

[68] Weinstein E.A., Ordonez A.A., Demarco V.P., Murawski A.M., Pokkali S., Macdonald E.M., Klunk M., Mease R.C., Pomper M.G., Jain S.K. (2014). Imaging Enterobacteriaceae infection in vivo with 18F-fluorodeoxysorbitol positron emission tomography. Sci. Transl. Med..

[69] Miller L., Winter G., Baur B., Witulla B., Solbach C., Reske S., Lindén M. (2014). Synthesis, characterization, and biodistribution of multiple 89Zr-labeled pore-expanded mesoporous silica nanoparticles for PET. Nanoscale.

[70] Rahmani S., Shahhoseini S., Mohamadi R., Vojdani M. (2017). Synthesis, Quality Control and Stability Studies of 2-[18F]Fluoro-2-Deoxy-D-Glucose(18F-FDG) at Different Conditions of Temperature by Physicochemical and Microbiological Assays. Iran. J. Pharm. Res. IJPR.

[71] Martiniova L., De Palatis L., Etchebehere E., Ravizzini G. (2016). Gallium-68 in Medical Imaging. Curr. Radiopharm..

[72] Ordonez A.A., Weinstein E.A., Bambarger L.E., Saini V., Chang Y.S., Demarco V.P., Klunk M.H., Urbanowski M.E., Moulton K.L., Murawski A.M. (2016). A Systematic Approach for Developing Bacteria-Specific Imaging Tracers. J. Nucl. Med..

[73] Li J., Zheng H., Fodah R.A., Warawa J.M., Ng C.K. (2017). Validation of 2-18F-Fluorodeoxysorbitol as a Potential Radiopharmaceutical for Imaging Bacterial Infection in the Lung. J. Nucl. Med..

[74] Moek K.L., Giesen D., Kok I.C., De Groot D.J.A., Jalving M., Fehrmann R.S., Hooge M.N.L.-D., Brouwers A.H., De Vries E.G. (2017). Theranostics Using Antibodies and Antibody-Related Therapeutics. J. Nucl. Med..

[75] Schoenmakers J.W.A., Heuker M., López-Álvarez M., Nagengast W.B., Van Dam G.M., Van Dijl J.M., Jutte P.C., Van Oosten M. (2020). Image-guided in situ detection of bacterial biofilms in a human prosthetic knee infection model: A feasibility study for clinical diagnosis of prosthetic joint infections. Eur. J. Nucl. Med. Mol. Imaging.

[76] Lau J.S.Y., Kiss C., Roberts E., Horne K., Korman T.M., Woolley I.J. (2017). Surveillance of life-long antibiotics: A review of antibiotic prescribing practices in an Australian Healthcare Network. Ann. Clin. Microbiol. Antimicrob..

[77] Lau J.S.Y., Korman T.M., Woolley I. (2018). Life-long antimicrobial therapy: Where is the evidence?. J. Antimicrob. Chemother..

[78] Perng P., Marcus C., Subramaniam R.M. (2015). (18)F-FDG PET/CT and Melanoma: Staging, Immune Modulation and Mutation-Targeted Therapy Assessment, and Prognosis. AJR Am. J. Roentgenol..

[79] Mayr A., Aigner M., Lass-Flörl C. (2011). Anidulafungin for the treatment of invasive candidiasis. Clin. Microbiol. Infect..

